# Install, repair and outcomes: a special issue on physiological and programmed DNA lesions

**DOI:** 10.3724/abbs.2022058

**Published:** 2022-06-15

**Authors:** Fei-Long Meng

**Affiliations:** State Key Laboratory of Molecular Biology Shanghai Institute of Biochemistry and Cell Biology Center for Excellence in Molecular Cell Science Chinese Academy of Sciences University of Chinese Academy of Sciences Shanghai 200031 ChinaE-mail: Feilong.Meng@sibcb.ac.cn

DNA lesions are by-products of various cellular activities, and the resulting genome instability can lead to deleterious outcomes, including tumorigenesis. However, as the ancient Chinese philosopher Lao Tzi noted, misfortune may be a blessing in disguise. Programmed and/or physiological DNA lesions could result in beneficial outcomes. In this context, the antigen receptor diversification in adaptive immunity, first described in the 1980s, serves as an example of beneficial somatic DNA alteration. In recent years, emerging evidence has accumulated on the install, repair, and outcomes of physiological/programmed DNA lesions. This special issue includes 11 reviews from researchers in this particular field to present the latest updates.

Programmed DNA lesions can be introduced through particular enzymes, including DNA nuclease and DNA modification enzymes. In the mammalian adaptive immune system, the RAG endonuclease can cut the DNA in the antigen receptor gene loci, while activation-Induced cytidine deaminase (AID) further introduces DNA modification in the loci to diversify the DNA sequences. In a review by Yu [1], the biochemical features of AID are summarized and a working model of how AID works on the various physiological substrates is proposed. The review presents an excellent example of the sophisticated regulation of a mutator enzyme for the general audience. Another convergently evolved adaptive immune system, CRISPR/Cas9, has recently been developed into a powerful tool for gene editing. Yin and Hu [2] introduce the progress of this gene-editing tool with a focus on the sources of unwanted by-products. Our readers will have a comprehensive sight on the development and challenge of this technique based on programmed DNA lesions.

The programmed DNA lesions activate the general cellular DNA repair pathways and are channeled into different products in an either error-free or error-prone manner. In a review by Dong and colleagues [3], the error-prone alternative end-joining (Alt-EJ) pathway is thoroughly discussed in the context of antigen receptor gene recombination and oncogenic chromosomal translocations. Compared to the DNA strand breaks, DNA modifications (e.g. base damage, crosslinks, and mispairing) occur more frequently under the physiological condition. Wang *et al*. [4] review the research progress of the base excision repair (BER) pathway and present the molecular basis for a key enzyme, DNA glycosylase, in this pathway. Similarly, Zhang *et al*. [5] depict the nucleotide excision repair (NER) pathways in handling DNA damages either caused by environmental toxins or originating from physiological activities. With the exampled DNA repair pathways, our readers should get a glimpse of how the general DNA repair pathways are harnessed to repair the programmed/physiological lesions.

The adaptive immunity is achieved through the repair of programmed DNA lesions. In the past four decades, the molecular pathways underlying immune diversity have been well-documented. However, emerging concepts continue to arise in the field, e.g., the generation of rare broadly neutralizing antibodies with special sequence characteristics. Feng *et al*. [6] discuss the possible mechanisms that generate insertions and deletions, long complementarity determining region 3, and high frequencies of mutations. Furthermore, cellular DNA repair factors have recently been demonstrated to participate in innate immunity. Mao and colleagues [7] summarize the up-to-date progress in the intersection field of DNA repair and innate immunity. In the current Coronavirus Disease 2019 (COVID-19) pandemic, the DNA repair deficient patients showed increase susceptibility to SARS-CoV-2 infections. Wang *et al*. [8] summarize the pathogenic mechanisms and clinical manifestations of COVID-19 and propose a model of intensified innate immune response in this special patient population. Besides the immune system, physiological/programmed DNA lesions play critical roles in various biological systems, including the hematopoiesis and neuronal development. Li *et al*. [9] review the sources of DNA lesions and unique characteristics of DNA repair in hematopoietic stem cells, which are also discussed in the context of human genetic disorders. Along the same vein, Zheng [10] summarizes the unique strategies and regulators in pluripotent stem cells and the implication in clinical applications of stem cells in regenerative medicine.

Discovery in the physiological/programmed DNA lesion field is driven by the state-of-the-art technologies, including mathematical models, high-throughput sequencing based methods, single molecule techniques, and computational simulations. In this issue, the authors also showcase the new technologies applied in the field. Zhang *et al*. [11] describe the statistical principles underlying high-throughput sequencing of B cell receptor (BCR) repertoire and highlight the new analyses for our audience from biomedical sciences. Yin and Hu [2] depict their pipelines to detect the unwanted editing by-products of Cas9 cutting, and Zhang *et al*. [5] describe the methods to profile nucleotide excision repair. The application of these mathematical models and sequencing methods should inspire researchers to explore their favorite topics. Furthermore, Wang *et al* . [4] present an elegant application of computational simulation in the conformational dynamics study of DNA modifying enzymes. Aided with these interdisciplinary technologies, the field expects innovative discoveries in sources of physiological DNA lesions, unique repair mechanisms, and novel physiological roles in more biological processes.
